# Clinical characteristics, complications, and predictive model of histological chorioamnionitis in women with preterm premature rupture of membranes

**DOI:** 10.1371/journal.pone.0283974

**Published:** 2023-04-06

**Authors:** Marco Aurélio Knippel Galletta, Regina Schultz, Mariana Fabbri Guazzelli de Oliveira Pereira Sartorelli, Eliane Cerqueira Leite Guerra, Isabela Karine Rodrigues Agra, Stela Verzinhasse Peres, Rossana Pulcineli Vieira Francisco

**Affiliations:** 1 Department of Obstetrics and Gynecology, Faculdade de Medicina, Universidade de São Paulo (FMUSP), São Paulo, SP, Brazil; 2 Division of Pathological Anatomy, Hospital das Clínicas da Faculdade de Medicina da Universidade de São Paulo (FMUSP), São Paulo, SP, Brazil; BC Children’s Hospital, CANADA

## Abstract

We aimed to analyze the impact of histological chorioamnionitis (HCA) in the presence of preterm premature rupture of the membranes (PPROM) on obstetric and neonatal outcomes, and its possible predictability. A retrospective cohort analysis of PPROM cases (20–37 weeks) was conducted comparing the patients with and without HCA, seeking a predictive model of HCA using logistic regression. A total of 295 cases of PPROM were selected, of which 72 (24.4%) had HCA. The group with HCA had a shorter latency period and a greater number of clinical and laboratory criteria in the evolution. The group with HCA had a worse comparative result and presented: lower gestational age at delivery, lower average birth weight, lower Apgar scores, longer neonatal hospitalization, worse maternal clinical conditions and, higher rates of stillbirth, low birth weight (LBW), very low birth weight (VLBW), complications in pregnancy and childbirth, and cesarean delivery due to fetal distress or chorioamnionitis. A predictive model for HCA was developed, with the following independent variables: abdominal pain (odds ratio [OR] = 11.61), uterine activity (noticeable contractions on physical exam) (OR = 5.97), fever (OR = 5.77), latency > 3 days (OR = 2.13), and C-reactive protein (OR = 1.01). With this model, an adequate receiver operating characteristic curve was found, with an area under the curve of 0.726, and some HCA probability curves were constructed for different clinical situations. In this novel study, we present a non-invasive predictive model, with clinical and laboratory variables, which may help in decision-making in a patient with PPROM.

## Introduction

Preterm premature rupture of membranes (PPROM) is a condition affecting 2–3% of pregnant women [1], and accounts for about a third (32.6%) and one fifth (18.2%) of preterm births in the US [2] and Brazil [3]. PPROM is considered the most common intercurrence in prematurity [4]. Treatment protocols vary greatly from one location to another [5] but are generally based on postponing childbirth with expectant management to reduce the inherent risk of prematurity. However, several complications may arise, one of the most serious and worrisome being intrauterine infection, or chorioamnionitis, which can negatively impact both maternal and neonatal prognoses.

The clinical diagnosis of chorioamnionitis is not always accurate, and the best marker of intrauterine infection remains Author. Therefore, the accurate identification of women with intrauterine infection continues to be a major challenge [6]. Accurate diagnosis of intrauterine infection is also important to determine the best time for delivery, balancing the benefits of prolonging the pregnancy, and the impact on perinatal morbidity and mortality associated with infection [7].

This condition has been recently described as intrauterine infection, inflammation, or both (triple I) [8] based on the inability to establish the most deleterious component of the disorder, with indications that the inflammatory component would lead to greater neonatal damage than the infectious component [9]. In addition, there is neither a degree of severity nor a perfect discrimination between clinical chorioamnionitis (CCA) and histological chorioamnionitis (HCA), with CCA cases not presenting a histological condition and vice versa [10]. Nevertheless, most researchers establish HCA as the best marker of fetal and neonatal impairment, despite the fact that this diagnosis is only verified days after delivery and at rates that can reach up to 60% of cases [11]. For this reason, many authors focus on describing CCA-related factors [12]. However, even CCA lacks unanimous diagnostic criteria [5, 13–15]. Nevertheless, identification of the intrauterine inflammatory and infectious environment appears important in determining optimal treatment for the newborn, not only regarding antibiotic therapy but also because these newborns may benefit differently from increased surfactant dose, restrictive use of invasive and prolonged ventilation, and postnatal administration of corticosteroids [12]. Therefore, early diagnosis of chorioamnionitis is important to facilitate differentiated treatment for the newborn before histological diagnosis, which, despite being the most critical component, can take several days to be obtained. To address this challenge, epidemiological or biological markers to detect chorioamnionitis before or immediately after birth should be investigated [12, 15].

According to some authors, HCA would be the best marker of severity in prematurity, as these situations being associated with serious consequences for the newborn, such as lower gestational age (GA), weight, and Apgar score at birth; higher rates of orotracheal intubation; greater use of surfactant; greater risk for retinopathy of prematurity, respiratory distress syndrome, and early-onset neonatal sepsis (EOS); longer hospital nursery stay [16], which may also be associated with increased risk for necrotizing enterocolitis, patent ductus arteriosus, intraventricular hemorrhage, and chronic lung disease [17–22].

Therefore, considering the lack of consistent data in the Brazilian scenario, and the absence of an accurate predictive model for this serious condition, we proposed to carefully analyze the available data of patients with PPROM and HCA at our hospital, with the aim of establishing a predictive model of HCA that can provide the identification of its best clinical and biochemical markers before birth.

## Methods

This retrospective cohort study included all consecutive PPROM cases admitted to the Obstetric Clinic of Hospital das Clínicas da Faculdade de Medicina da Universidade de São Paulo (HC-FMUSP), in São Paulo, SP, Brazil, between 2006 and 2011, with available data on delivery and GA at delivery of > 20 and < 37 weeks. We chose this gestational age period because this is the period in which we can propose conservative and expectant management. Although the gestational age between 20 and 26 weeks is for non-viable fetuses, there is a reasonable possibility of chorioamnionitis and maternal involvement, and the most adequate assessment of its risks can help in the conduction and orientation of the case. Furthermore, we chose this 5-year period because the protocol of the American Centers for Disease Control and Prevention (CDC) [23] started being used as of April 2011, and all PPROM patients started receiving antibiotic therapy, which could modify the results. The cases were selected from the computerized system of the HC-FMUSP Obstetric Clinic (Microsoft Access), with complete verification of the medical records and laboratory test results through the electronic platform of the Central laboratory of Hospital das Clínicas.

As it is a retrospective study which included reviewing of medical records, data were not always available for all patients.

Patients were treated according to the previously published HC-FMUSP obstetric clinic protocol [24, 25], with expectant management up to 36 weeks or resolution before that, in case of labor (not inhibited), abnormal fetal vitality, or chorioamnionitis. The diagnosis of CCA was made through this previously established protocol in the simultaneous presence of at least two of the following clinical or laboratory signs: fever (≥ 37.8 °C), with no other apparent focus; maternal tachycardia (heart rate > 100 bpm); fetal tachycardia (baseline heart rate > 160 bpm); uterine activity (irritable uterus with some clinically noticeable contractions or increased uterine sensitivity); purulent vaginal content, usually with a change in odor; leukocytosis (> 15,000 cells/mm^3^ or increase of 20%); increased C-reactive protein (CRP) by 20%; abrupt decrease in amniotic fluid index (AFI), usually greater than 50%; alteration of the fetal biophysical profile, with absence of fetal respiratory movements [26]. Regarding the fever data, it is important to emphasize that our care protocol stipulates fever when temperature is ≥ 37.8 °C [26]. This is a Brazilian characteristic, endorsed by the Ministry of Health [27], being different from the ACOG definition, which would be ≥ 38.0 °C [28].

Physical exam and fetal surveillance (with AFI and with fetal heart rate measurement) were performed daily. In the assessment of amniotic fluid, we considered oligohydramnios and sever oligohydramnios when AFI < 5 and < 3 [29, 30], respectively. Laboratory tests were performed every 48 h. Vital signs (maternal heart rate and temperature) were checked four times a day [26]. The present study evaluated only the presence of HCA diagnosed through histological analysis of the placenta and membranes after delivery, according to the technique established by the HC-FMUSP Pathological Anatomy Service.

HCA was diagnosed by acute maternal inflammatory reaction in the chorion and amnion, both in the ovular membranes and in the chorionic plate, which may be associated with fetal inflammatory reaction. The maternal reaction was characterized by polymorphonuclear neutrophils in the chorion laeve that migrate from the decidua parietalis to the chorion, or from the subchorionic intervillous space (maternal) to the chorion, moving toward the amnion. The diagnosis of fetal inflammatory reaction was made in the presence of fetal polymorphonuclear cells from umbilical vessels or chorionic arteries and veins in the chorion. The presence of an inflammatory reaction in the umbilical cord is called omphalitis or funisitis. This inflammation in the cord can affect only the umbilical vein, or the vein and arteries.

Demographic, clinical, and obstetric variables were taken from the patients’ charts. The various clinical comorbidities of pregnant women, such as heart disease, hypertension, nephropathy, asthma, rheumatological diseases, and others were grouped into a single category as "comorbidities." We separated two clinical categories due to the possible association with preterm labor and PPROM: "diabetes” and “urinary tract infection.” Miscarriage was defined as gestational loss at < 20 weeks, and cervical incompetence as "the inability of the uterine cervix to retain a pregnancy in the second trimester in the absence of clinical contractions, labor, or both" [31].

Finally, we sought to associate possible maternal and neonatal outcomes and complications with the presence or absence of HCA. As we have expectant management up to 36 weeks, we raised the possibility of terminating the pregnancy before that, either due to signs of fetal distress (alteration of the fetal biophysical profile or cardiotocography), premature labor, or chorioamnionitis. We also verified the presence of labor induction, type of delivery, and other maternal conditions at this moment. Regarding the newborn, we checked the gestational age at delivery, and divided the weight into low weight and very low weight, in addition to data from cord blood gases (when available); Apgar score at 1, 5, and 10 minutes after birth; and perinatal mortality. For the definition of a small-for-gestational-age (SGA) newborn, we consider the birth weight < 10th percentile of the Fenton & Kim curve [32].

The data were arranged in an Excel table and analyzed using the IBM SPSS 23 statistical software. Initially, HCA was descriptively analyzed with numerical variables as means, and standard deviations and categorical variables as frequencies, proportions, and percentages.

Patients with and without HCA were then compared using the chi-square test with Fisher’s correction for categorical variables, when appropriate. Normal distribution was confirmed for continuous numerical variables using the Kolmogorov–Smirnov test. All continuous numerical variables presented with p < 0.05 in normality tests; we therefore chose the Mann–Whitney U test for independent samples.

Following this analysis, we developed a predictive HCA model by listing the most significant variables in univariate analysis, with p < 0.100. We used stepwise forward logistic regression to estimate the odds ratio (OR) and their respective 95% confidence intervals (95%CI). Additionally, we produced a probability curve for the HCA event with the predictive factors observed in the logistic regression using the following log-linear function:

$$\log\left(\frac{pk}{p1}\right)=b0+b1*x1+b2*x2+\ldots +b20\;*\;x20,\text{with}\;k=\;2,3,4\ldots$$

The quality of the predictive model was verified using a receiver operating characteristic (ROC) curve, with area under the curve (AUC) calculation.

### Ethical consideration

The research project was approved by the Internal Research Project Approval Committee (CIAPP) of the Department and by the Research Project Approval Committee (CAPPesq) of Hospital das Clínicas under approval no. 248.040, of 4/17/2013 and registered on Plataforma Brasil under the no. CAAE 13978413.2.0000.0068. As this was a retrospective research project, the requirement for written informed consent was waived. Medical record analysis and patient identification were carefully conducted.

## Results

A total of 295 consecutive PPROM cases were selected for analysis. Of these, 72 exhibited HCA, representing a frequency of 24.4%. Of the 72 cases, 28 had omphalitis, with the infection reaching the fetus. This represented 9.5% of all PPROM cases and 38.9% of specific HCA cases.

Of the 72 HCA cases, 22 had a clinical diagnosis of chorioamnionitis before the resolution of the pregnancy. There were eight additional cases of CCA without HCA. Thus, the overall rate of CCA would be 30 in 295 cases or 10.17%.

Regarding the demographic characteristics and obstetric history, there was no statistically significant difference between the groups with and without HCA for most variables (race, age, place of birth, marital status, number of gestations, primiparous, comorbidities, diabetes, urinary infection, twinning, fetal malformation, and bleeding in the first half of pregnancy), except for the fact that HCA patients had prenatal care less frequently at the institution (44.4% vs. 63.8%, p = 0.005) with fewer appointments (average of 5.44 vs. 6.33, p = 0.038), in addition to a tendency to have lower education (< high school: 45% vs. 31.8%, p = 0.059) and higher frequency of repeated miscarriages (9.7% vs. 4%, p = 0.075) and cervical incompetence (8.6% vs. 3%, p = 0.063).

Table 1 shows the initial condition of these patients on admission. The group that developed HCA had lower GA at the time of rupture of the membranes and on admission, with an apparent higher frequency of bleeding in the initial condition, a difference that was not statistically significant.

**Table 1:** Initial clinical condition of 295 pregnant women with PPROM according to the presence or absence of HCA.

Variable	With HCA N (%) or Mean (SD)	Without HCA N (%) or Mean (SD)	p-value
Typical history	57/72 (79.2%)	132/171 (77.2%)	0.735 †
Bleeding on admission	11/69 (15.9%)	14/165 (8.5%)	0.092 †
Diagnostic doubt	9/72 (12.5%)	35/172 (20.3%)	0.146 †
GA at complaint	30.51 (4.903)	33.32 (4.065)	0.000 ‡ *
GA on admission	30.76 (4.539)	33.48 (3.784)	0.000 ‡ *
BMI on admission	28.50 (5.587)	29.35 (5.886)	0.328 ‡
Weight on admission (kg)	71.89 (14.679)	75.13 (15.58)	0.126 ‡
Obesity (BMI > 30 kg/m^2^) on admission	28/44 (63.6%)	70/118 (59.3%)	0.617 †
Initial AFI	7.47 (4.315)	8.95 (7.235)	0.340 ‡
Initial oligoamnion (AFI < 5)	11/42 (26,2%)	21/80 (26,2%)	0.994 †
Initial leukocyte count	11,469.3 (4,096.2)	10,851.2 (2,527.27)	0.548 ‡
Initial CRP	20.17 (28.610)	12.81 (15.590)	0.380 ‡

Abbreviations: HCA = histological chorioamnionitis; GA = gestational age; BMI = body mass index; AFI = amniotic fluid index; CRP = C-reactive protein. Statistics:

† Chi-Square;

‡ Mann-Whitney Test;

* p < 0.05.

Table 2 compares patients with PPROM progressing to HCA with the other patients. Almost 2/3 of these women had a clinical diagnosis of chorioamnionitis as they fulfilled at least two of the necessary criteria for diagnosis in the service. The latency period was on-average shorter than the one of patients who did not have HCA, being longer than three days for half of the HCA group. Although the initial amniotic fluid index (AFI) was not related to the onset of HCA, a lower final AFI was related to HCA, particularly when it was < 3. However, an abrupt AFI decrease i.e., the criterion used in our service for the clinical diagnosis was not related to the presence of HCA. The absence of respiratory movement (RM), another clinical criterion used in the service, was also not related to the presence of HCA. However, a fetal biophysical profile with a lower score was related to HCA occurrence. CRP and leukocyte count were not associated with the presence of HCA on admission, rather only at the final evaluation. CRP that changed at some point in time was not associated with the development of HCA; only a higher mean final value, and a difference between the last two and between the first and last tests were associated. The same was true for leucocyte count, which was also associated with the presence of HCA when it changed (> 15,000) at some point in the follow-up. Although fever was related to HCA, this occurred in < 15% of pregnant women and the maximum temperature on the day of delivery was not related to the presence of HCA; only the mean maximum temperature at some point in the follow-up was associated. On the other hand, both maternal (> 100 bpm) and fetal tachycardia (≥ 160 bpm) were related to the occurrence of HCA. However, only the mean maternal heart rate was associated with the development of HCA. Finally, two clinical criteria were truly relevant, being present in most pregnant women with HCA: uterine pain and the presence of contractions.

**Table 2:** Characteristics of the clinical and laboratory progression of 295 pregnant women with PPROM according to the presence or absence of Histological Chorioamnionitis.

Variable	With HCA N (%) or Mean (SD)	Without HCA N (%) or Mean (SD)	P
Latency period	8.16 (16.00)	9.30 (20.37)	0.005 ‡ *
Latency > 3 days	35/70 (50%)	64/176 (36.4%)	0.049 † *
Latency ≥ 5 days	30/70 (42.9%)	60/176 (34.1%)	0.198 †
Number of chorioamnionitis criteria	2.43 (1.419)	0.49 (0.803)	0.000 ‡ **
Presence of 2 or more criteria	39/54 (72.2%)	12/128 (9.4%)	0.000 ‡ **
Corticosteroid use	7/53 (13.2%)	12/128 (9.4%)	0.444 †
Corticosteroid repetition	1/72 (1.4%)	1/177 (0.6%)	0.496 §
Use of any antibiotic	19/52 (36.5%)	34/123 (27.6%)	0.242 †
AFI < 3	15/41 (36.6%)	14/85 (16.5%)	0.012 † *
Final AFI	5.506 (4.067)	7.674 (4.434)	0.006 ‡ *
Final AFI < 5	16/35 (45.7%)	24/76 (31.6%)	0.150 †
Difference Ini-Fin AFI	-1.574 (3.717)	-1.513 (8.150)	0.229 ‡
Difference final 2 AFI	-0.139 (3.261)	-0,826 (3.270)	0.772 ‡
Final FBP	8.228 (2.314)	9.333 (1.303)	0.004 ‡ *
Absent BM	4/40 (10%)	3/85 (3.5%)	0.209 §
Changed CRP (> 5)	25/36 (69.4%)	34/47 (72.3%)	0.773 †
Final CRP	49.19 (53.655)	15.20 (22.291)	0.000 ‡ **
Difference Ini-Fin CRP	39.28 (51.951)	3.19 (13.458)	0.000 ‡ **
Difference Ini-Fin CRP %	870.67 (1,794.80)	40.02 (157.75)	0.000 ‡ **
Difference final 2t CRP	36.23 (48.488)	-1.31 (12.15)	0.000 ‡ **
Difference last CRP %	582.43 (1,638.27)	20.73 (112.93)	0.000 ‡ **
Leukocytes > 15 000	30/67 (44.8%)	19/135 (14.1%)	0.000 † **
Final leukocyte count	14,345.5 (5,498.0)	11,198.7 (3,493.9)	0.000 ‡ **
Difference Ini-Fin Leuko	3,150.5 (6,185.9)	665.8 (4,193)	0.000 ‡ **
Difference Ini-Fin Leuko %	37.78 (62.179)	-1.055 (26.888)	0.000 ‡ **
Difference final 2 Leuko	3,529.8 (4,751.7)	503.6 (2,020)	0.000 ‡ **
Difference final 2 Leuko %	33.10 (50.914)	6.58 (18.402)	0.001 ‡ *
Max temp	37.1 (0.594)	36.7 (0.415)	0.006 ‡ *
Temp delivery day	36.72 (0.783)	36.81 (3.858)	0.087 ‡
Fever (≥ 37.8 °C)	7/48 (14.6%)	1/122 (0.82%)	0.001 § *
Max MHR	99.92 (11.56)	93.13 (13.55)	0.003 ‡ *
MHR delivery Day	93.57 (13.15)	82.69 (10.29)	0.000 ‡ **
Tachycardia (MHR > 100 bpm)	26/51 (51%)	19/111 (17.1%)	0.000 † **
Basal final MHR	141.69 (39.816)	145.34 (8.305)	0.641 ‡
Fetal tachycardia (FHR ≥ 160 bpm)	10/39 (25.6%)	6/95 (6.3%)	0.002 † **
Uterine pain	26/50 (52%)	10/120 (8.3%)	0.000 † **
Presence of contractions	42/49 (85.7%)	69/121 (57%)	0.000 † **

Table 3 shows that the presence of HCA on anatomopathological examination was associated with a more severe clinical condition, with a higher rate of neonatal and maternal complications. Newborns presented with lower GA at delivery, LBW, and higher rates of LBW, VLBW, and extremely LBW (ELBW), as well as changed Apgar scores at 1, 5, and 10 minutes. Despite this, no umbilical cord changes were observed in pH monitoring; perhaps because only 29 of the newborns with HCA and 75 of those without HCA underwent this assessment. However, this condition may explain not only a longer hospital stay in the nursery (means of 34 x 13 days,), but also a higher rate of perinatal mortality, due to stillbirths.

**Table 3:** Childbirth, newborn, and postpartum progression data of 295 women with PPROM, according to the presence or absence of HCA.

Variable	With HCA N (%) or Mean (DP)	Without HCA N (%) or Mean (DP)	OR (95%CI)	p-value
GA at delivery	31.48 (4.355)	34.58 (2.74)		0.000 ‡ **
Newborn weight	1,794.36 (744.80)	2,317.27 (618.63)		0.000 ‡ **
Days of maternal hospitalization	7.90 (5.243)	9.91 (16.083)		0.010 ‡ *
Cesarean delivery	32/72 (44.4%)	104/177 (58.8%)	0.56 (0.32–0.97)	0.040 † *
Resolution by premature labor	44/72 (61.1%)	96/177 (54.2%)	1.33 (0.76–2.31)	0.322 †
Labor induction	8/71 (11.3%)	33/176 (18.8%)		0.153 †
Labor time	4.75 (3.544)	4.79 (4.205)		0.858 ‡
Complications during childbirth	30/72 (41.7%)	64/177 (36,2%)		0.416 †
Resolution by fetal distress	8/72 (11.1%)	10/177 (5.6%)	1.88 (0.76–2.32)	0.187 †
Indication of Cesarean section for fetal distress	14/72 (19.4%)	16/177 (9%)	2.43 (1.11–5.30)	0.022 † *
Resolution by chorioamnionitis	10/72 (13.9%)	5/177 (2.8%)	5.55 (1.82–16.87)	0.002 § *
Unpleasant odor at birth	20/63 (31.7%)	5/161 (3.1%)	14,51 (5.17–40.91)	0.000 § **
Good clinical conditions at birth	51/72 (70.8%)	155/177 (87.6%)		0.002 † *
Regular general status at childbirth	21/72 (29.2%)	22/177 (12.4%)	2.90 (1.47–5.71)	0.002 † *
Presence of any complications in pregnancy and childbirth	30/72 (41.7%)	47/177 (26.6%)	1.97 (1.11–3.51)	0.019 † *
Postpartum ICU	6/51 (11.8%)	6/130 (4.6%)	2.76 (0.84–8.98)	0.082 ‡
LBW (< 2,500 g)	56/70 (80%)	101/175 (57.7%)	2.93 (1.52–5.65)	0.001 † **
VLBW (< 1,500 g)	23/70 (32.9%)	19/175 (10.9%)	4.02 (2.02–8.00)	0.000 † **
ELBW (< 1,000 g)	15/70 (21.4%)	7/175 (4%)	6.54 (2.54–16.88)	0.000 † **
SGA newborn	9/70 (12.9%)	35/175 (20%)	0.59 (0.2701.30)	0.188 †
Female newborn	36/69 (52.2%)	81/175 (46.3%)		0.407 †
Apgar 1st min	5.59 (3.187)	7.71 (2.282)		0.000 ‡ **
Apgar 5th min	7.40 (3.196)	8.79 (2.127)		0.000 ‡ **
Apgar 10th min	7.91 (3.220)	9.11 (2.133)		0.000 ‡ **
Apgar 1st min < 7	34/70 (48.6%)	28/175 (16%)	4.96 (2.67–9.21)	0.000 † **
Apgar 5th min < 7	15/70 (21.4%)	12/175 (6.9%)	3.70 (1.63–8.40)	0.001 † **
Apgar 10th min < 7	12/70 (17.1%)	10/174 (5.7%)	3.39 (1.39–8.27)	0.005 † *
Umbilical cord pH	7.202 (0,124)	7.220 (0.085)		0.764 ‡
Acidosis (pH < 7.20)	11/29 (37.9%)	29/75 (38.7%)		0.945 †
Umbilical cord pO_2_	21.448 (11.793)	20.272 (8.476)		0.994 ‡
Umbilical cord pCO_2_	59.33 (18.553)	56.81 (12.763)		0.755 ‡
Bicarbonate	22.03 (3.003)	22.22 (3.220)		0.251 ‡
Perinatal death	12/70 (17.1%)	16/176 (9.1%)	2.07 (0.92–4.63)	0.073 †
Stillbirth	6/70 (8.6%)	5/176 (2.8%)		0.050 † *
Died after birth	6/70 (8.6%)	11/176 (6.3%)		0.517 †
Days of nursery hospitalization	34.29 (39.31)	13.00 (22.28)		0.012 ‡ *

Abbreviations: OR (95%CI) = odds ratio (95% confidence interval); HCA = histological chorioamnionitis; ICU, intensive care unit; LBW, low birth weight newborn; VLBW, very low birth weight newborn; ELBW, extremely low birth weight newborn; SGA, small for gestational age. Statistics:

† Chi-Square;

‡ Mann–Whitney Test;

§ Fisher;

* p < 0.05;

** p < 0.001

As for the mother, the cesarean section rate was lower, mainly due to spontaneous preterm labor, with a shorter hospital stay, probably related to the shorter latency period. However, the rate of fetal distress was higher, particularly when cesarean section was indicated. This explains a higher frequency of parturients in a regular general condition and a lower frequency of those in a good general condition, with a higher rate of complications (including puerperal infection, endometritis, reoperation, renal function worsening, hemodynamic disorders, placental debris retention, cardiac decompensation, and acute anemia) in the immediate postpartum. There was only one case of confirmed sepsis in the HCA group, but there were no cases of hysterectomy or maternal death. In addition, HCA patients had a higher ICU stay but without statistical significance.

Logically, chorioamnionitis clinically diagnosed in HCA patients had a higher rate of resolution but it is noteworthy that only 10 of the 72 HCA cases were recognized as such before delivery, indicating resolution. An unpleasant odor was present in 20 of these cases at the time of delivery, confirming chorioamnionitis.

Next, significant variables or with marginal significance in univariate analysis (p < 0.100) were selected for multivariate analysis, trying to group those that were similar. The following variables were selected through binary logistic regression for the HCA outcome: education level < high school, latency > 3 days, presence of fever (≥ 37.8 °C) at some point, fetal tachycardia (> 160 bpm), uterine pain, irritable uterus, GA at complaint, final AFI, final CRP, final leukocyte count, percentage difference in the last two leukocyte counts, percent difference in the last two CRPs, maternal heart rate on the day of delivery, and maximum temperature.

This selection of variables was used for a stepwise forward regression, and we obtained an appropriate model, with a Hosmer and Lemeshow Test of 0.672, leaving four significant and independent variables in the final model, as shown in Table 4. Independent factors associated with HCA were presence of uterine pain, maximum temperature, uterine activity, and final CRP adjusted for the latency period (> 3 days). Pregnant women with uterine pain had an OR = 16.3 (95% CI: 3.23–82.52). Each increase of 1.79 °C in maximum temperature generated an OR = 6.01 (95 95%: 1.2–30.4). Pregnant women with uterine activity were six-fold more likely to have an HCA outcome. Each mean increase of 0.023 mg/L in the final CRP generated an OR = 1.02 (95% CI: 1.01–1.04). All the results of the predictive model are presented in Table 4.

**Table 4:** Predictive model of HCA obtained through forward conditional binary logistic regression, controlled for latency > 3 days.

	B	S.E.	OR	OR (CI95%)		Sig.
				Lower	Upper	
Abdominal pain (Y/N)	2.452	.731	11.611	2.770	48.670	0.001
Uterine activity (Y/N)	1.788	.802	5.976	1.241	28.778	0.26
Fever (Y/N)	1.753	1.062	5.773	.721	46.241	0.099
Latency > 3 days (Y/N)	.756	.710	2.130	.530	8.558	0.287
Final CRP	.026	.008	1.027	1.011	1.042	0.001
Constant	-3.985	.988	.019			0.000

Fever: ≥ 37.8°C. Abbreviations: HCA = histological chorioamnionitis; CRP = C-reactive protein; B = beta; OR = odds ratio (Exp B); 95% CI = 95% confidence interval, with lower and higher values; Sig. = significance level; S.E. = standard error

An ROC curve was constructed with this predictive model to evaluate the sensitivity of the method in predicting HCA, with an AUC = 0.726 (95% CI: 0.627–0.825) and p < 0.001.

**Fig 1:**
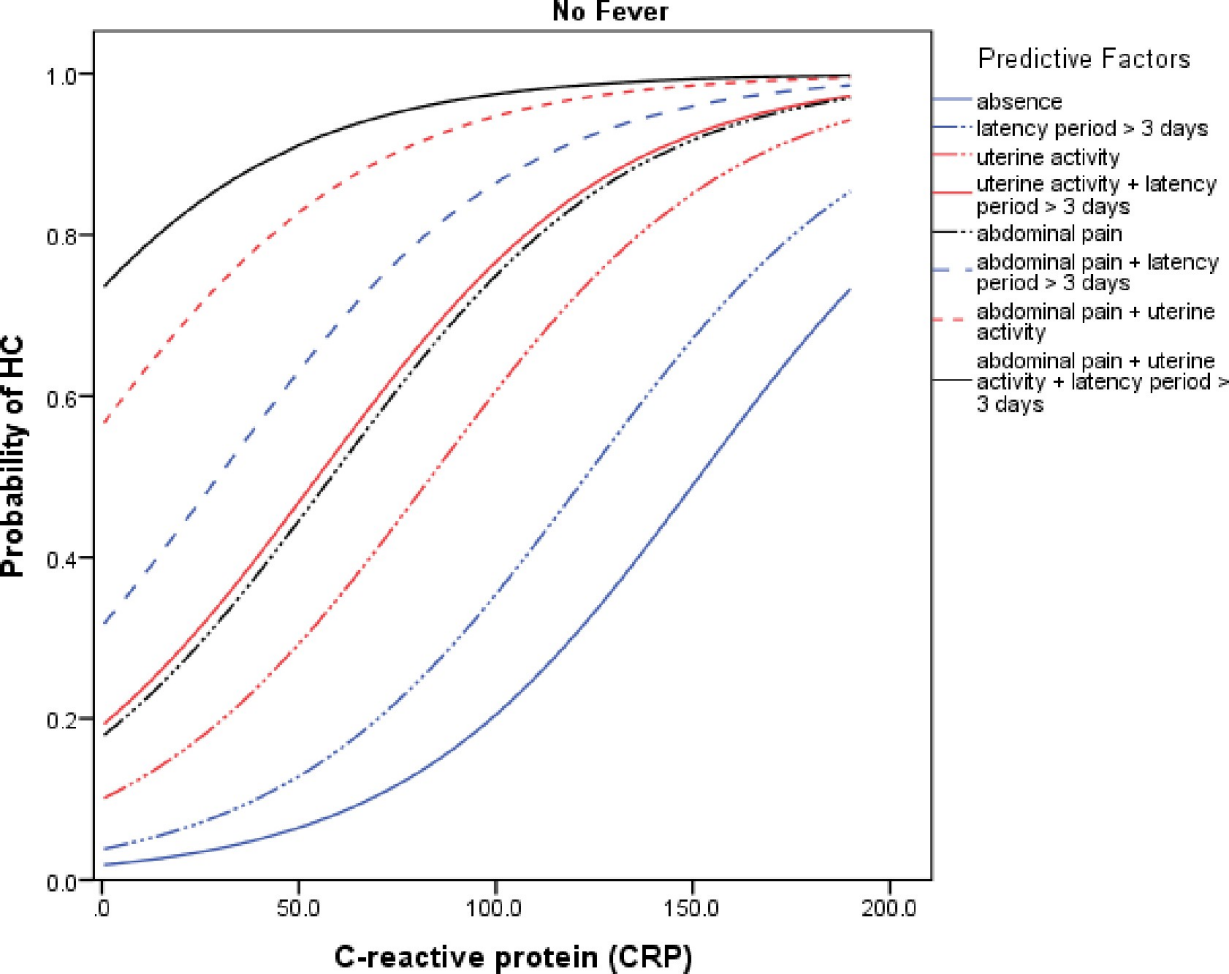
Probability curve of the HCA event according to CRP levels in the presence of various clinical conditions including maternal fever (≥ 37.8 °C).

**Fig 2:**
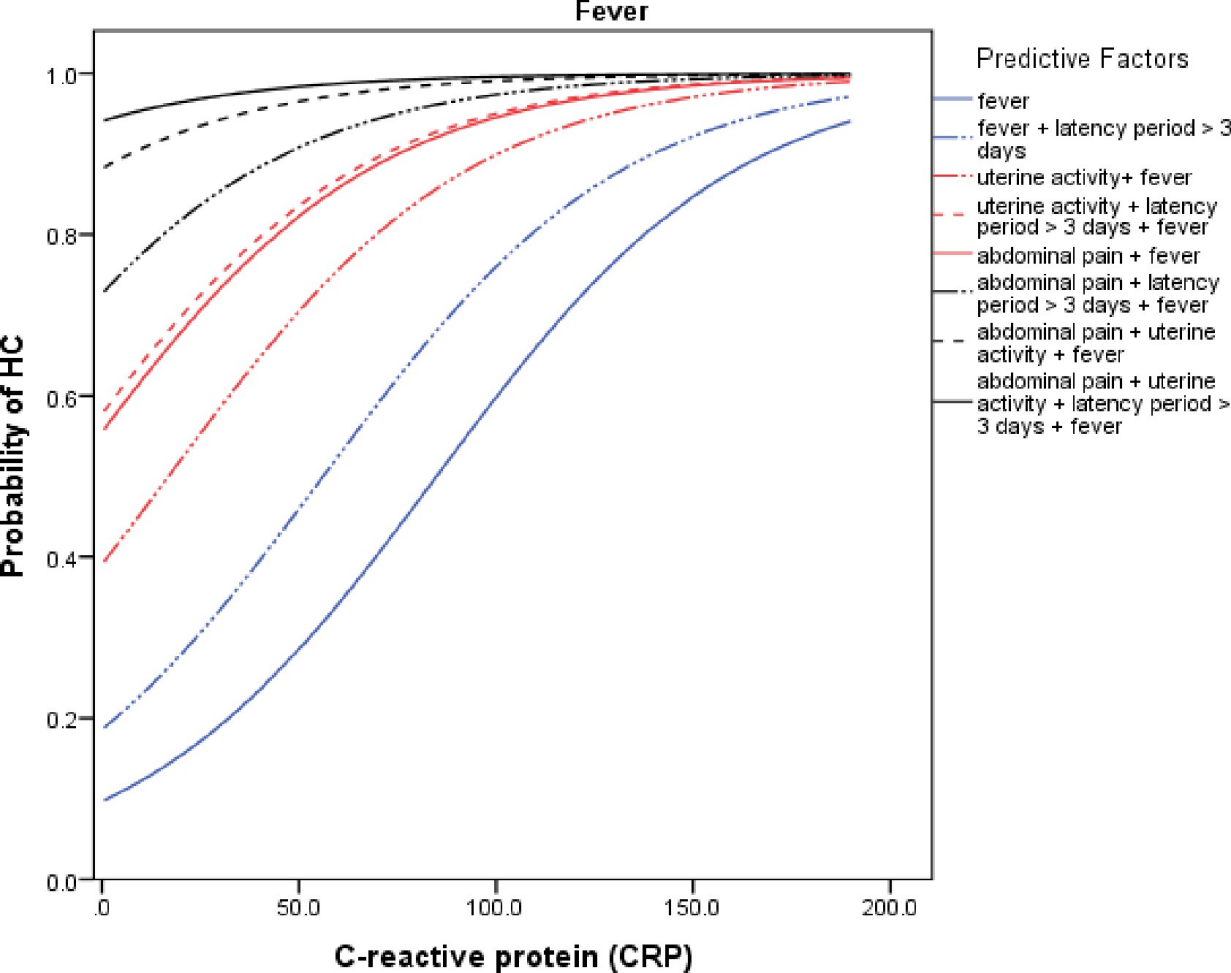
Probability curve of the HCA event according to CRP levels in the presence of various clinical conditions without maternal fever (< 37.8 °C).

To assess the probability of the HCA event, we constructed two probability curves according to CRP variation and considering the main clinical factors of the final predictive model. In the first and second curve, we considered the presence (Fig 1) and absence of fever (Fig 2), respectively.

## Discussion

Our rate of HCA (24.4%) was not remarkably high, particularly considering that all cases in the study were premature with a mean GA of 30.7 weeks on admission. A French multicenter study similar to ours analyzed 295 pregnant women with GA between 22 and 37 weeks and reported a HCA rate of 42.7% [33]. A Korean article reported a HCA rate of 41.6% in 149 pregnant women with GA between 20 and 37 weeks [34]. A study from Lithuania involving 137 pregnant women with PPROM at < 34 weeks showed a HCA rate of 37.95% [35], whereas a Turkish study [36] on previable PPROM (14–24 weeks) reported a CCA rate of 24.48%, similar to our HCA rate. A study by Ryan et al. [16] in Ireland involving newborns aged < 32 weeks also demonstrated a similar HCA rate of 25.4%, but with only 30.6% PPROM. The international literature reports HCA ranging from 0.6% [37] to 57.7% [38] in patients with PPROM. This great variation could be related to several factors, including the type of study; prospective studies report higher rates than retrospective studies. The population investigated also presents different prevalence of risk factors, diagnostic criteria, and changes in obstetric practice based on time and between centers. Further, the incidence increases with decreasing GA [39].

In Brazil, there are few studies evaluating chorioamnionitis as a complication of PPROM. We identified only three, but with different GA compared to ours. A study of patients with GA < 24 weeks reported a CCA and HCA rates of 71% and 36%, respectively [40]. Another study, involving cases with GA between 28 and 34 weeks, reported an HCA rate of 80% [41]. A third study with subjects at GA < 34 weeks, exhibited a rate of 67.1% for CCA and HCA combined [42].

Other Brazilian studies show higher rates of HCA than ours, ranging from 26.9% [43] to 40% [44], but with different populations not restricted to patients with PPROM [44]. Finally, another Brazilian study analyzed chorioamnionitis from the pathologist’s point of view, and reported only 15% PPROM in 90 cases of histological inflammation in the placenta and membranes at different GAs but with a predominance of term pregnancies (72.2%), without reference to HCA-specific prevalence data [45].

Therefore, our present study appears to be the first to evaluate this association and its prediction in Brazil.

The diagnosis of CCA is not always easy. Often the diagnosis is made only retrospectively. The clinical picture is not always clear, and the diagnostic criteria are not uniform, even in Brazil. Although the ACOG does not establish unpleasant odor among its chorioamnionitis criteria [28], we use it in our daily clinical practice [26]. Interestingly, we found a statistically significant association between this criterion and HCA. But we were careful to consider this variable only during childbirth, as the odor is often not noticed before this moment. Perhaps for this very reason, this variable did not remain independent in the final predictive model. A challenge to be considered in the future would be to standardize this criterion in a more objective way.

Indeed, there is debate about the best definition of chorioamnionitis because the underlying inflammatory condition is often not diagnosed in CCA. HCA would be easier, but it is only possible retrospectively, following birth. Bastek et al. [46] demonstrated that most (87%) of patients with CCA also have HCA but only 22.4% of those with HCA were clinically diagnosed. Interestingly, in the group with certain adverse neonatal outcomes, HCA (60.1%) was more frequent than CCA (19%), i.e., HCA increased the risk of adverse neonatal outcomes more than seven-fold (OR: 7.18; 4.98–10.34). A study by Smulian et al. [10] also corroborate this difficulty in comparing HCA with CCA. Most (61.9%) of patients with CCA also had HCA, but the histological examination of the placenta was not consistent in one third of the cases with clinical diagnosis. In 13.7% of cases, there was no recorded objective description of chorioamnionitis and most of these patients (57.9%) had histological evidence of chorioamnionitis. Kim et al. [11] reported CCA and HCA rates of 8.9 and 50.7%, respectively, in Korea. Of the 74 and 13 patients with HCA and CCA, 10 each also had CCA and HCA, respectively. Our data revealed a similar situation, with 74% of patients with CCA also having HCA, whereas only 31% of patients with HCA also had CCA.

In a review on the subject, Newton [39] also highlighted the fact that histological evidence of placental inflammation may not always be associated with microbiological evidence. Amniotic fluid or membrane cultures fail to document bacterial infection in about a quarter of placentas with HCA. Negative cultures can occur in HCA, either due to inappropriate microbiological techniques for certain organisms (such as Mycoplasma) or due to the previous administration of antibiotics.

Therefore, we decided to study HCA, with the understanding that it would constitute an appropriate marker of worse prognosis in PPROM. Indeed, our data support this assertion as they represent a more serious condition when HCA is associated with PPROM with a shorter latency period; lower GA at delivery; lower newborn weight; and higher rates of LBW, VLBW, fetal distress, unpleasant odor, delivery complications, low Apgar score at 1, 5, and 10 min, and stillbirth. Although maternal hospitalization was shorter, newborn hospitalization was longer, demonstrating a more severe condition and perinatal predominance (fetal and neonatal). This corroborates with findings from some authors who report that HCA is worse than CCA [10, 11, 16, 33, 46–49, 51].

Indeed, several authors indicated worse neonatal indices in patients with HCA, such as lower GA, LBW [10, 11, 16, 33, 47, 49–51], and low Apgar scores [10, 16, 49–51], in addition to higher rates of complications such as respiratory distress syndrome [16, 48], EOS [10, 16, 47, 51, 52], retinopathy of prematurity [16, 48, 51], intracranial bleeding [16, 33, 53], bronchopulmonary dysplasia [1], and necrotizing enterocolitis [48].

Such an increase in the risk of several neonatal complications should obviously be associated with a higher risk of perinatal death. Data from Han et al. [52] complements ours, with a higher risk of perinatal death in pregnant women with HCA. They reported eight perinatal deaths (seven at birth and one following), resulting in a frequency of 2.4% in patients with HCA versus 0.3% in controls, with a 7-fold increased risk of perinatal death (OR: 7.14; 2.49–20.47). Based on these results, these authors encourage the histological evaluation of all placentas of preterm births, concluding that HCA would be associated with more perinatal comorbidities and possess greater diagnostic importance than CCA.

However, if HCA is more severe, would we be able to identify it before delivery? Unfortunately, the initial condition of our patients on admission did not establish a clear difference between patients who would progress with or without HCA other than a lower GA at PROM and on admission. Furthermore, there was only a non-significant trend toward more bleeding on admission i.e., 15.9% vs. 8.5% (p = 0.09) for HCA and CCA, respectively. However, we observed a progressive clinical condition associated with HCA, with higher rates of severe oligohydramnios (AFI < 3); lower final AFI; high final CRP with a significant increase between the last two tests (582% vs. 20%); higher rates of leukocytosis with difference between the last two tests (33.1% vs. 6.5%) but not as significant as that with CRP; fever at some point but not necessarily on the day of delivery; a slightly higher maximum temperature (37.1 vs. 36.7 °C). There were also higher rates of maternal or fetal tachycardia, in addition to a higher mean maternal heart rate at some point or even on the day of delivery. The higher frequency of uterine contractions and lower frequency of abdominal pain are noteworthy. These signs are all considered in the clinical diagnosis of chorioamnionitis but they were not always present at the same time and to the extent expected; otherwise, the rate of CCA would be similar to that of HCA.

Such changes in the clinical course are corroborated by other authors. Smulian et al. [10] described the presence of fever in 61.9% of patients, fetal tachycardia in 50.4%, maternal tachycardia (> 100 bpm) in 45.3%, odorous amniotic fluid in 7.4%, and sensitive uterus in 5.3%, in addition to a higher leukocyte count in the final test (13, 900 vs. 11, 000). Of the cases without fever, 56.6% had at least one clinical finding consistent with chorioamnionitis. Caloone et al. [33] described higher rates of changed fetal heart rate (44.6% vs. 30.2%) and leukocytosis (36.5% vs. 23.6%) but similar rates of maternal tachycardia (23.8% vs. 23.07%), uterine contractions (73.8% vs. 69.2%), and hyperthermia (2.38% vs. 2.36%).

A British study [54] at the King’s College Hospital School of Medicine in London, identified intrauterine infection through amniocentesis in 35.7% of the investigated patients with few maternal symptoms although intrauterine contamination had already occurred. They reported tachycardia in 14% (> 100 bpm), fever in 7% (≥ 38 °C), leukocytosis in 14% (> 15,000), and high CRP in 28% (> 2 mg/dL), which appeared to be more strongly associated with the event [45].

Ryan et al. [16] also described the important role of CRP level in HCA, with a CRP > 5 mg/L in 93.7% of cases. These findings corroborated the data by Popowski et al. [55] from two French hospitals. They assessed 399 women with PPROM at > 34 weeks and more than 72 h in the service, and described a HCA and CCA rate of 10.8% and 5.3%, respectively, emphasizing the important role of CRP in HCA and EOS. The analysis of the predictive model for the HCA event showed a ROC curve with an AUC of 0.62 for serum CRP dosage. The curve for the diagnosis of EOS also included serum leukocyte count in the model, with a more significant ROC and AUC of 0.82. However, using CRP alone (≥ 5 mg/L), a similar AUC (0.80) was maintained, with slightly better predictive values and a slightly higher OR (14.7 vs. 12.0), indicating that CRP alone could be used to predict these cases.

Our data describe a different predictive model, in which not only CRP but other clinical variables were associated. In fact, few authors have made a predictive model of HCA in pregnant women with PPROM like we did, with clinical and laboratory variables in a non-invasive way. Only two authors built predictive models with clinical and laboratory variables. However, one of them applied to pregnant women with and without PPROM [18], and the other only applied to preterm patients with PPROM [56].

Ma et al. [56] conducted a study, retrospectively analyzing data from 69 Chinese women with PROM, of whom 22 were diagnosed with HCA (31.88%). Three predictive models were developed for HCA, the first one with CRP, which provided an AUC of 0.651; the second was designed with a Lab-Score, which involved the platelet/leukocyte ratio and serum triglyceride levels in addition to CRP. This model improved the ROC curve, with an AUC of 0.724. The third model included clinical criteria, involving the Beck Depression Inventory (BDI), diastolic blood pressure, and the presence or absence of prematurity. With this model, the prediction improved and resulted in an AUC of 0.828 when the other criteria were included [56].

Been et al. [18] evaluated 216 women, of whom 60 (27.7%) had PPROM, and established a predictive model for HCA in which, in the absence of SGA or preeclampsia, the following factors would be independently associated: PPROM (OR = 1.02), GA ≤ 28 weeks (OR = 1.34), and CCA (OR = 1.64).

The other authors only describe predictive models in the laboratory. Two of these authors [11, 49] made their predictive model with laboratory variables invasively via amniocentesis, that is, in a way that would not be reproducible in most of our hospitals still counting on the risk of amniocentesis. And, finally, three authors [33, 50, 57] made a predictive model for HCA in PPROM with only laboratory variables, with markers in maternal blood with a CRP predominance.

Cobo et al. [49] analyzed amniotic fluid by amniocentesis from 107 Czech pregnant women with PPROM, and established a final predictive model for HCA with IL-6 as the single independent variable, with an AUC of 0.78 and an accuracy of 71%.

Kim et al. [11] retrospectively evaluated 146 pregnant Korean women with PPROM between 20 and 33 weeks 6/7 days using laboratory tests and amniocentesis. They created two predictive models: In the first model with non-invasive variables, the initial GA remained, with an OR of 4.90 and a serum CRP level > 5.0 mg/L, with an OR of 2.612. This model resulted in a significant ROC curve with AUC of 0.742. The second model with invasive variables (from amniocentesis) recognized IL-6 levels in the amniotic fluid as a significant predictive variable, with an OR of 3.557 and an AUC of 0.757.

Stepan et al. [50] studied 386 pregnant women with PPROM in the Czech Republic and built a predictive model for HCA and bacterial invasion of the amniotic cavity. The best marker for this condition was CRP level, with a cutoff level of 6.45 mg/L providing an AUC of 0.66 in pregnancies < 32 weeks.

Perrone et al. [57] performed a retrospective study with 66 Italian pregnant women with PPROM, including 35 with HCA (53%) and 24 with funisitis (36.3%), with HCA present in 20/24 of the patients with funisitis. The authors created a predictive model for funisitis considering it would be a more reliable marker of intra-amniotic infection, and produced a ROC curve with an AUC of 0.671 for CRP on admission which improved to an AUC of 0.737 when considering the CRP level prepartum.

Caloone et al. [33] collected several serum markers in France. The analysis of the different ROC curves showed that the most predictive marker for HCA was the CRP, with an AUC of 0.7 (95% CI: 0.64–0.77), followed by IL-6, with an AUC of 0.63, and by matrix-metalloproteinase 9 (MMP-9), with an AUC of 0.62, and MMP-8, with an AUC of 0.61, in addition to human neutrophile peptides (HNP), with an AUC of 0.58. Our study resulted in an interesting predictive model for combining typical clinical variables such as abdominal pain, uterine activity, latency period, and the presence of fever with CRP as a laboratory variable easily available in peripheral blood in almost all Brazilian hospitals. Many studies highlight the relevant role of this inflammatory marker in predicting and diagnosing HCA but they do not relate it to clinical variables as we did here. Studies analyzed other inflammatory markers together, improving the predictive value of the model. However, the feasibility of these models is not good, since such markers are obtained by amniocentesis, and are still expensive and not available in the daily practice of most hospitals. The predictive model created in this study has an adequate ROC curve with an AUC of 0.726, which is higher than that for most predictive models.

PPROM is a distressing clinical situation for both patients and obstetricians. It is not easy to balance the risk of prematurity on the one hand and the risk of intrauterine infection on the other. The lower the GA, the more difficult the decision to deliver or maintain the pregnancy. We believe that the results of this study can collaborate in the daily decision-making process of obstetricians who will be able to consider the risk of chorioamnionitis more precisely, based on not only laboratory but also on clinical data. This will provide a more adequate balance between the risk of infection and of prematurity. In this sense, evaluating the probability curves may help in the decision-making process at the bedside. Although this study is carried out in Brazil where it is unprecedented in its considerations, we believe that its results can help obstetricians worldwide. In conclusion, the present study is relevant not only in the Brazilian scenario, but also internationally, as its approach is unprecedented in considering clinical and laboratory results easily available on a daily basis in the prediction of a serious condition such as HCA. However, this predictive model currently requires validation in new samples of patients with PPROM due to greater antibiotic and corticosteroid use. We will be evaluating this in our new research project.
